# Non-linear association of the metabolic score for insulin resistance with obstructive sleep apnea: a cross-sectional study from NHANES 2015–2018

**DOI:** 10.3389/fnut.2025.1545140

**Published:** 2025-02-26

**Authors:** Zhimao Cai, Jiachen Li, Hui Peng, Ye Ye, Sixia Chen, Lingli Zeng, Jiashuang Lin, Weifeng Chen

**Affiliations:** ^1^Department of General Medicine, Shenzhen Second People’s Hospital, The First Affiliated Hospital of Shenzhen University, Shenzhen, Guangdong, China; ^2^Shenzhen University Health Science Center, Shenzhen, Guangdong, China; ^3^Department of Orthopedics, Shenzhen Second People’s Hospital, The First Affiliated Hospital of Shenzhen University, Shenzhen, Guangdong, China; ^4^Shantou University Medical College, Shantou, Guangdong, China

**Keywords:** obstructive sleep apnea, metabolic score for insulin resistance, insulin resistance, NHANES, cross-sectional study

## Abstract

**Background:**

The relationship between the Metabolic Score for Insulin Resistance (METS-IR), a novel index integrating multiple metabolic parameters, and the risk of obstructive sleep apnea (OSA) remains under explored.

**Methods:**

Analyses were conducted on data from 2,348 participants included in the National Health and Nutrition Examination Survey (NHANES) data from 2015 to 2018. Logistic regression, stratified analyses, curve-fitting analyses, and threshold effects analyses were employed to evaluate the association between METS-IR and the risk of OSA.

**Results:**

Multifactorial logistic regression analyses revealed a significant positive correlation between METS-IR and the risk of OSA [OR: 1.05 (95% CI: 1.04–1.06)]. Stratified analyses showed consistent associations across various subgroups, including sex, race, age, marital status, education level, poverty income ratio, physical activity, alcohol use, smoking status, diabetes mellitus, hypertension, and cardiovascular disease. Nonlinear analysis identified an inflection point at METS-IR 46.65. On the left of the inflection point, the risk of OSA increased significantly, with each unit increase in METS-IR associated with a 7% increase in risk [OR: 1.07 (95% CI: 1.05–1.08)]. On the right side of the inflection point, however, the rate of risk increase slowed to 1% [OR: 1.01 (95% CI: 1.00–1.02)].

**Conclusion:**

This investigation reveals a significant and nonlinear relationship between METS-IR and OSA. Further investigation is needed to explore their association more comprehensively and to elucidate the underlying mechanisms.

## Introduction

Obstructive sleep apnea (OSA) is a frequently encountered sleep disorder characterized by intermittent upper airway obstruction during sleep, which results in hypoxia and sleep disturbances (1). Extensive studies have confirmed a strong link between OSA and multiple chronic conditions, particularly cardiovascular, metabolic, and cerebrovascular diseases (2, 3). Evidence shows that metabolic syndrome (4), which includes obesity (5), hypertension (6), diabetes mellitus (7), and hyperlipidemia (8), markedly elevates the likelihood of developing OSA. Among these, obesity is particularly influential, as the accumulation of adipose tissue within the upper airway can precipitate airway obstruction; additionally, OSA has the potential to aggravate metabolic disorders, engendering a harmful cycle (9, 10).

Furthermore, there exists a strong association between OSA and insulin resistance (11), a critical pathological element in metabolic conditions such as non-alcoholic fatty liver disease (NAFLD) and type 2 diabetes mellitus (12). Insulin resistance promotes a reciprocal interaction between OSA and metabolic disorders through mechanisms such as sympathetic nervous system activation, oxidative stress, and systemic inflammation (13, 14). Given the complex interrelations between OSA and metabolic dysfunction, it is imperative to detect and intervene in OSA risk factors early to prevent further disease progression.

The Metabolic Insulin Resistance Score (METS-IR) is a newly formulated, non-invasive index that provides a reliable estimate of insulin resistance by incorporating fasting glucose levels, triglyceride concentrations, and body mass index (BMI) (15). METS-IR has demonstrated efficacy in predicting various metabolic diseases, including hypertension, NAFLD, and cardiovascular conditions (16, 17). However, there is a scarcity of comprehensive studies exploring the link between METS-IR and OSA. Considering the shared pathophysiological mechanisms linking insulin resistance and OSA, examining the connection between METS-IR and OSA could provide critical insights for both diagnosis and therapeutic approaches.

The objective of this research was to analyze the link between METS-IR and OSA. This study sought to clarify the potential role of METS-IR as a predictive marker for obstructive sleep apnea (OSA) by analyzing a nationally representative dataset, thereby providing a scientific foundation for developing targeted intervention strategies for at-risk populations.

## Methods

### Study design and population

**Figure 1:**
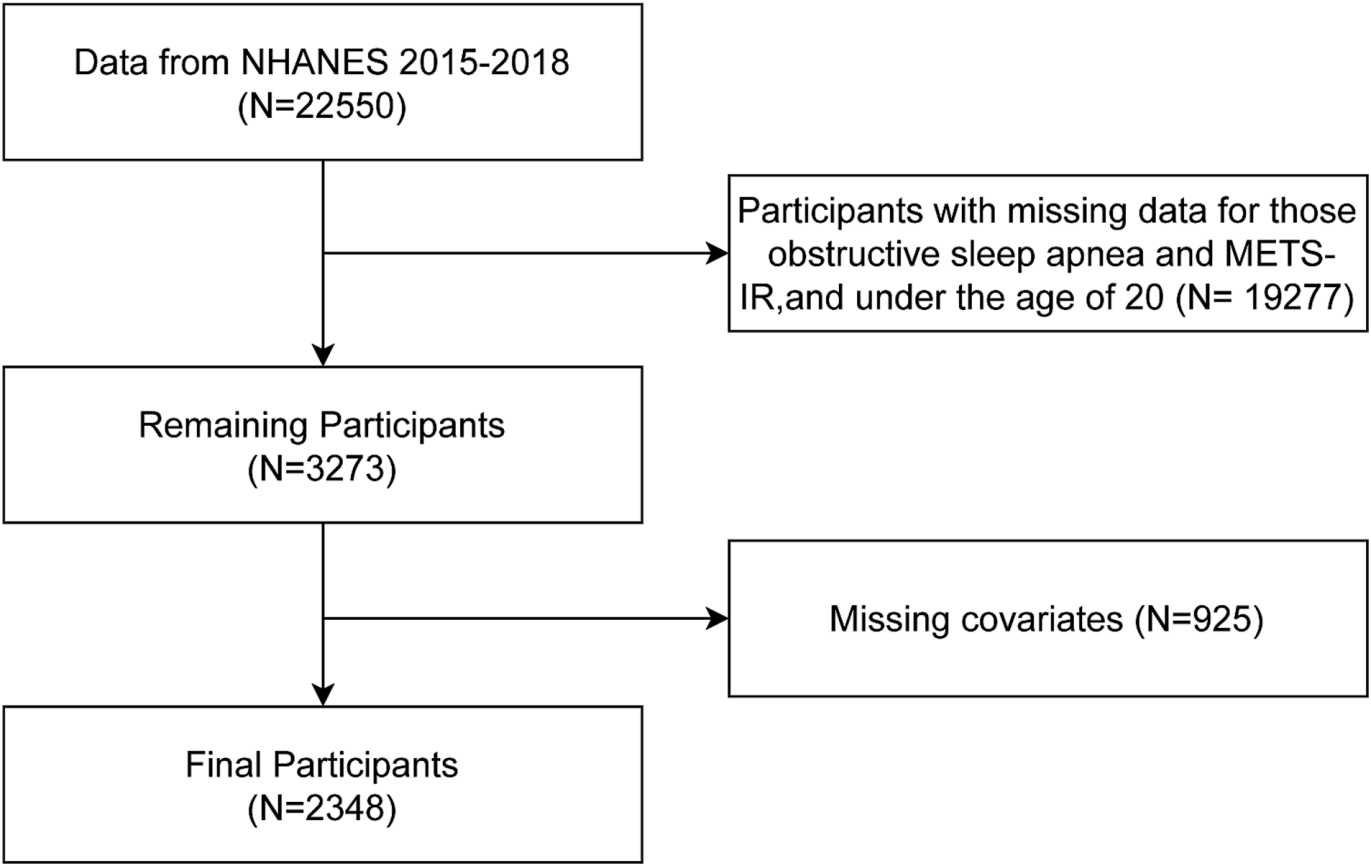
Flow chart of participants selection.

This study’s dataset was derived from the National Health and Nutrition Examination Survey (NHANES), encompassing data collected between 2015 and 2018.Established by the National Center for Health Statistics (NCHS), which operates under the Centers for Disease Control and Prevention, the NHANES program is designed to assess the health and nutritional conditions of the general U.S. civilian population. Detailed information about the program, including data collection methods and available data files, can be found at http://www.cdc.gov/nchs/nhanes.html. This study was conducted in compliance with the ethical standards set by the NCHS Ethics Review Board, with each NHANES survey cycle obtaining the necessary clearance. Participant consent was secured at the recruitment stage, ensuring the ethical integrity of the research. In this research, we strictly followed the guidelines of the Strengthening the Reporting of Observational Studies in Epidemiology (STROBE) for the reporting of cross-sectional observational studies. The research leveraged data from NHANES 2015–2018, comprising a participant pool of 22,550 individuals. Exclusion criteria included: individuals with incomplete records regarding OSA and METS-IR, those below the age threshold of 20 years, and subjects with incomplete covariate data. After exclusions, the study encompassed a refined group of 2,348 participants for analysis, as delineated in Figure 1.

### Assessment of the OSA

Diagnoses of OSA were based on participants^’^ responses to a questionnaire specifically designed to evaluate sleep habits and disorders (18). Participants were classified as having OSA if they indicated “yes” to any of the following criteria: (1) experiencing snoring three or more times per week; (2) having wheezing, snoring, or episodes of breathing cessation on at least three nights per week; (3) feeling excessively sleepy during the day 16–30 times a month, even after sleeping seven or more hours each night.

### Measurement of METS-IR

The METS-IR was identified as a principal variable within the context of this study. The formula employed for calculating METS-IR is defined as follows (19):


$$\mathrm{METS}-IR=Ln\left(2\times FBG+TG\right)\times BMI∕Ln\;\left(HDL-C\right)$$

Fasting glucose and triglyceride levels were measured enzymatically using automated biochemical analyzers, namely the Roche Cobas 6000 and Roche Modular P systems. These tools guarantee accurate quantification of serum triglyceride levels. Deriving the body mass index (BMI) entails the division of an individual’s weight in kilograms by the square of their height in meters. Participant data regarding weight and height were sourced from the Examination Data within the “Body Measure” section.

### Study covariates

The identification of covariates was informed by a synthesis of prior research and theoretical models. This study pinpointed various demographic and health-related elements as potential covariates. Physical activity (PA) was evaluated based on whether participants engaged in walking or cycling. Smoking status was classified according to total lifetime cigarette consumption, with individuals having smoked 100 or more cigarettes categorized as smokers. The term “alcohol consumption” was defined as the consumption of 4/5 or more drinks per day. Hypertension was recognized as a diagnosis provided by a licensed healthcare professional. The diagnosis of diabetes mellitus was based on clinical identification by a qualified healthcare provider. Cardiovascular disease was identified through diagnoses of coronary artery disease, congestive heart failure, myocardial infarction, angina, or stroke, all confirmed by a qualified medical professional. Participants affirmatively reporting these health conditions were classified as having hypertension, diabetes mellitus, or cardiovascular disease. Evaluations of health status were derived from both self-reported data and assessments conducted by healthcare professionals regarding hypertension, diabetes mellitus, and cardiovascular disease.

### Statistical analysis

Statistical analyses were performed using EmpowerStats (version 4.2)^1^ and R software (version 4.3.2).^2^ Continuous variables were expressed as means with 95% confidence intervals (95% CIs) or medians with interquartile ranges, while categorical variables were represented as counts and proportions. Further evaluations utilized multivariable logistic regression and linear models to assess the influence of METS-IR on obstructive sleep apnea (OSA). The analysis without adjustments was termed the “crude model,” while Model 1 incorporated adjustments for gender, race, and age. Model 2 included additional controls for factors such as marital status, education level, poverty income ratio (PIR), physical activity (PA), alcohol intake, diabetes mellitus, smoking status, hypertension, and cardiovascular disease (CVD). Subgroup analyses and interaction tests were also conducted to explore potential variations across different populations. Non-linear associations between OSA and METS-IR were examined using smoothing spline fitting techniques. A *p*-value of less than 0.05 was considered statistically significant for all outcomes assessed.

## Results

### Baseline characteristics

In terms of baseline characteristics, the final sample for this study comprised 2,348 participants, with 50.11% identifying as male and 49.89% as female. The analysis indicated that the METS-IR was significantly elevated in the fourth quartile (Q4), suggesting a considerable prevalence of insulin resistance in this subset. A significant portion of the participants identified as Non-Hispanic White; nevertheless, notable racial disparities were observed across the quartiles (*p* = 0.0146). Individuals in the top quartile of METS-IR showed a higher prevalence of conditions associated with insulin resistance. Key factors contributing to this trend included older age (≥51.70 years), decreased high-density lipoprotein (HDL) levels, elevated triglycerides (TG), and increased fasting blood glucose (FBG) concentrations. In contrast, participants with lower METS-IR scores were generally younger, displaying higher HDL levels alongside reduced triglyceride and fasting blood glucose concentrations. The distribution of body mass index (BMI) across the quartiles exhibited significant variation (*p* < 0.0001), with a greater prevalence of individuals with a BMI of 30 or higher in the upper quartile. When comparing the highest quartile to the lowest, there was a markedly higher incidence of hypertension, diabetes mellitus, and cardiovascular disease (CVD) (*p* < 0.0001 for all), emphasizing a significant link between insulin resistance and these comorbidities (Table 1).

**Table 1:** Basic characteristics of participants in the METS-IR quartiles.

Variable	Overall	METS-IR quartiles				
		Q1(19.87–34.61)	Q2(34.62–42.33)	Q3(42.34–51.67)	Q4(51.70–131.37)	*p*-value
Age (year)	47.09 (45.90, 48.28)	43.46 (41.60, 45.31)	49.65 (47.49, 51.80)	47.78 (45.03, 50.52)	47.95 (46.12, 49.78)	<0.0001
FBG (mg/dL)	109.38 (107.62, 111.15)	97.99 (96.94, 99.03)	104.65 (102.86, 106.43)	111.20 (107.26, 115.13)	124.93 (120.32, 129.53)	<0.0001
HDL (mg/dL)	54.91 (53.67, 56.15)	69.12 (66.66, 71.58)	57.42 (55.35, 59.48)	48.23 (47.10, 49.37)	43.18 (41.97, 44.39)	<0.0001
TG (mg/dL)	114.22 (108.67, 119.77)	68.43 (64.80, 72.06)	96.54 (90.37, 102.71)	130.05 (117.92, 142.19)	167.01 (149.75, 184.27)	<0.0001
BMI (%)						<0.0001
<18.5	1.55 (1.02, 2.33)	5.67 (3.69, 8.62)	0.00 (0.00, 0.00)	0.00 (0.00, 0.00)	0.00 (0.00, 0.00)	
≥18.5, < 25.0	26.06 (23.55, 28.73)	79.92 (74.90, 84.14)	16.60 (11.73, 22.96)	0.96 (0.27, 3.40)	0.00 (0.00, 0.00)	
≥25.0, < 30.0	31.34 (28.74, 34.06)	14.42 (10.84, 18.92)	72.95 (66.99, 78.18)	38.67 (33.26, 44.37)	2.08 (1.03, 4.18)	
≥30.0	41.06 (38.09, 44.09)	0.00 (0.00, 0.00)	10.46 (7.07, 15.20)	60.37 (54.44, 66.01)	97.92 (95.82, 98.97)	
METS-IR	43.71 (42.86, 44.56)	29.64 (29.32, 29.96)	38.38 (38.13, 38.62)	46.48 (46.23, 46.73)	61.87 (60.90, 62.84)	<0.0001
Gender (%)						<0.0001
Male	50.11 (47.29, 52.93)	37.47 (32.46, 42.75)	53.68 (47.82, 59.44)	59.32 (54.62, 63.87)	51.68 (44.60, 58.71)	
Female	49.89 (47.07, 52.71)	62.53 (57.25, 67.54)	46.32 (40.56, 52.18)	40.68 (36.13, 45.38)	48.32 (41.29, 55.40)	
Race (%)						0.0146
Mexican American	8.75 (6.21, 12.19)	5.30 (3.46, 8.03)	8.31 (5.43, 12.50)	10.67 (6.83, 16.30)	11.13 (7.70, 15.82)	
Other Hispanic	5.89 (4.63, 7.47)	4.67 (3.10, 6.98)	6.45 (4.85, 8.52)	7.39 (4.49, 11.92)	5.25 (3.55, 7.69)	
Non-Hispanic White	66.85 (62.44, 70.97)	70.10 (64.67, 75.01)	67.28 (60.71, 73.23)	63.56 (56.97, 69.67)	66.02 (60.10, 71.48)	
Non-Hispanic Black	9.53 (7.20, 12.51)	8.49 (6.27, 11.39)	9.88 (6.77, 14.20)	9.55 (6.83, 13.19)	10.32 (6.95, 15.08)	
Other Race	8.99 (7.09, 11.33)	11.45 (8.42, 15.38)	8.09 (5.24, 12.30)	8.84 (5.57, 13.73)	7.28 (5.43, 9.69)	
Marital status (%)						0.0086
Married	55.17 (50.69, 59.57)	50.13 (43.56, 56.68)	53.98 (48.59, 59.28)	54.70 (46.81, 62.36)	62.40 (55.46, 68.86)	
Single	27.70 (24.48, 31.16)	27.61 (23.39, 32.27)	30.49 (24.03, 37.84)	32.37 (25.68, 39.86)	20.50 (16.23, 25.55)	
With partner	17.13 (14.91, 19.61)	22.26 (17.85, 27.39)	15.52 (10.88, 21.66)	12.93 (8.30, 19.60)	17.10 (12.76, 22.53)	
Education level (%)						0.4381
Under high school	89.64 (87.79, 91.24)	91.74 (88.82, 93.94)	89.20 (84.72, 92.48)	87.85 (82.92, 91.51)	89.49 (85.55, 92.45)	
High school or	10.36 (8.76, 12.21)	8.26 (6.06, 11.18)	10.80 (7.52, 15.28)	12.15 (8.49, 17.08)	10.51 (7.55, 14.45)	
PIR (%)						0.0130
<1.0	11.62 (9.97, 13.49)	8.51 (6.08, 11.77)	12.49 (9.46, 16.31)	12.26 (9.64, 15.47)	13.58 (9.81, 18.50)	
1.0–3.0	37.43 (33.89, 41.11)	34.78 (28.16, 42.03)	33.68 (28.02, 39.86)	44.82 (39.25, 50.51)	36.87 (30.24, 44.03)	
>3.0	50.96 (47.00, 54.90)	56.72 (49.35, 63.80)	53.82 (46.84, 60.66)	42.92 (37.35, 48.68)	49.55 (42.01, 57.11)	
PA						0.0025
No	77.91 (75.13, 80.45)	72.83 (67.55, 77.53)	75.05 (68.72, 80.46)	80.19 (74.83, 84.64)	84.13 (80.11, 87.46)	
Yes	22.09 (19.55, 24.87)	27.17 (22.47, 32.45)	24.95 (19.54, 31.28)	19.81 (15.36, 25.17)	15.87 (12.54, 19.89)	
Alcohol use (%)						0.3082
No	83.94 (81.69, 85.97)	86.42 (82.47, 89.59)	84.06 (79.92, 87.48)	83.38 (78.86, 87.09)	81.62 (77.13, 85.40)	
Yes	16.06 (14.03, 18.31)	13.58 (10.41, 17.53)	15.94 (12.52, 20.08)	16.62 (12.91, 21.14)	18.38 (14.60, 22.87)	
Smoking status (%)						0.8389
No	52.60 (49.08, 56.08)	54.63 (48.94, 60.21)	52.10 (46.07, 58.07)	52.09 (45.83, 58.29)	51.31 (45.06, 57.53)	
Yes	47.40 (43.92, 50.92)	45.37 (39.79, 51.06)	47.90 (41.93, 53.93)	47.91 (41.71, 54.17)	48.69 (42.47, 54.94)	
Diabetes mellitus (%)						<0.0001
No	90.10 (88.52, 91.49)	98.68 (97.87, 99.18)	94.21 (91.80, 95.95)	87.72 (83.67, 90.87)	78.87 (75.13, 82.19)	
Yes	9.90 (8.51, 11.48)	1.32 (0.82, 2.13)	5.79 (4.05, 8.20)	12.28 (9.13, 16.33)	21.13 (17.81, 24.87)	
Hypertension (%)						<0.0001
No	68.68 (65.39, 71.78)	83.67 (78.94, 87.50)	69.99 (63.83, 75.50)	63.94 (57.29, 70.09)	55.36 (49.81, 60.77)	
Yes	31.32 (28.22, 34.61)	16.33 (12.50, 21.06)	30.01 (24.50, 36.17)	36.06 (29.91, 42.71)	44.64 (39.23, 50.19)	
CVD (%)						0.0006
No	91.71 (89.75, 93.32)	94.61 (91.49, 96.63)	92.61 (88.67, 95.25)	92.49 (90.08, 94.35)	86.84 (82.19, 90.41)	
Yes	8.29 (6.68, 10.25)	5.39 (3.37, 8.51)	7.39 (4.75, 11.33)	7.51 (5.65, 9.92)	13.16 (9.59, 17.81)	
OSA (%)						<0.0001
No	50.10 (46.81, 53.39)	71.12 (65.32, 76.30)	52.54 (46.81, 58.19)	42.34 (36.30, 48.62)	31.93 (26.80, 37.55)	
Yes	49.90 (46.61, 53.19)	28.88 (23.70, 34.68)	47.46 (41.81, 53.19)	57.66 (51.38, 63.70)	68.07 (62.45, 73.20)	

FBG, fasting blood glucose; HDL, high-density lipoprotein; TG, triglycerides; BMI, body mass index; METS-IR, metabolic score for insulin resistance; PIR, poverty income ratio; PA, physical activity; CVD, cardiovascular disease; OSA, obstructive sleep apnea.

### Association between METS-IR and risk for OSA

The multivariable logistic regression analysis indicated a statistically significant positive link between METS-IR and the risk of OSA, which was consistent across all analytical models (*p* < 0.05). Following adjustments for all covariates, each additional unit of METS-IR was linked to a 5% increase in the prevalence of obstructive sleep apnea (OSA) among the participants (OR = 1.05, 95% CI 1.04–1.06). This trend remained stable across the three models evaluated. Participants within the top quartile of METS-IR experienced nearly a fivefold increase in OSA prevalence relative to those in the lowest quartile (OR = 4.95, 95% CI 3.32–7.37) (Table 2).

**Table 2:** Association between METS-IR and the risk of OSA.

Exposure	Crude model	Model 1	Model 2
	OR (95%CI) *P*-value	OR (95%CI) *P*-value	OR (95%CI) *P*-value
METS-IR	1.05 (1.04, 1.06) <0.0001	1.05 (1.04, 1.06) <0.0001	1.05 (1.04, 1.06) <0.0001
METS-IR quartile			
Q1 (19.87–34.61)	Reference	Reference	Reference
Q2 (34.62–42.33)	2.22 (1.60, 3.09) 0.0001	1.95 (1.41, 2.68) 0.0006	1.95 (1.39, 2.74) 0.0033
Q3 (42.34–51.67)	3.35 (2.34, 4.81) <0.0001	2.97 (2.09, 4.23) <0.0001	2.96 (2.07, 4.23) 0.0001
Q4 (51.70–131.37)	5.25 (3.58, 7.69) <0.0001	4.80 (3.34, 6.91) <0.0001	4.95 (3.32, 7.37) <0.0001
P for trend	<0.0001	<0.0001	0.0001

Model specifications are as follows: the crude model is unadjusted; Model 1 incorporates adjustments for sex, race, and age; whereas Model 2 extends these adjustments to include marital status, education level, poverty income ratio (PIR), physical activity (PA), alcohol consumption, smoking habits, as well as the presence of diabetes mellitus, hypertension, and cardiovascular disease.

### Analysis of curve fitting and threshold effects

**Figure 2:**
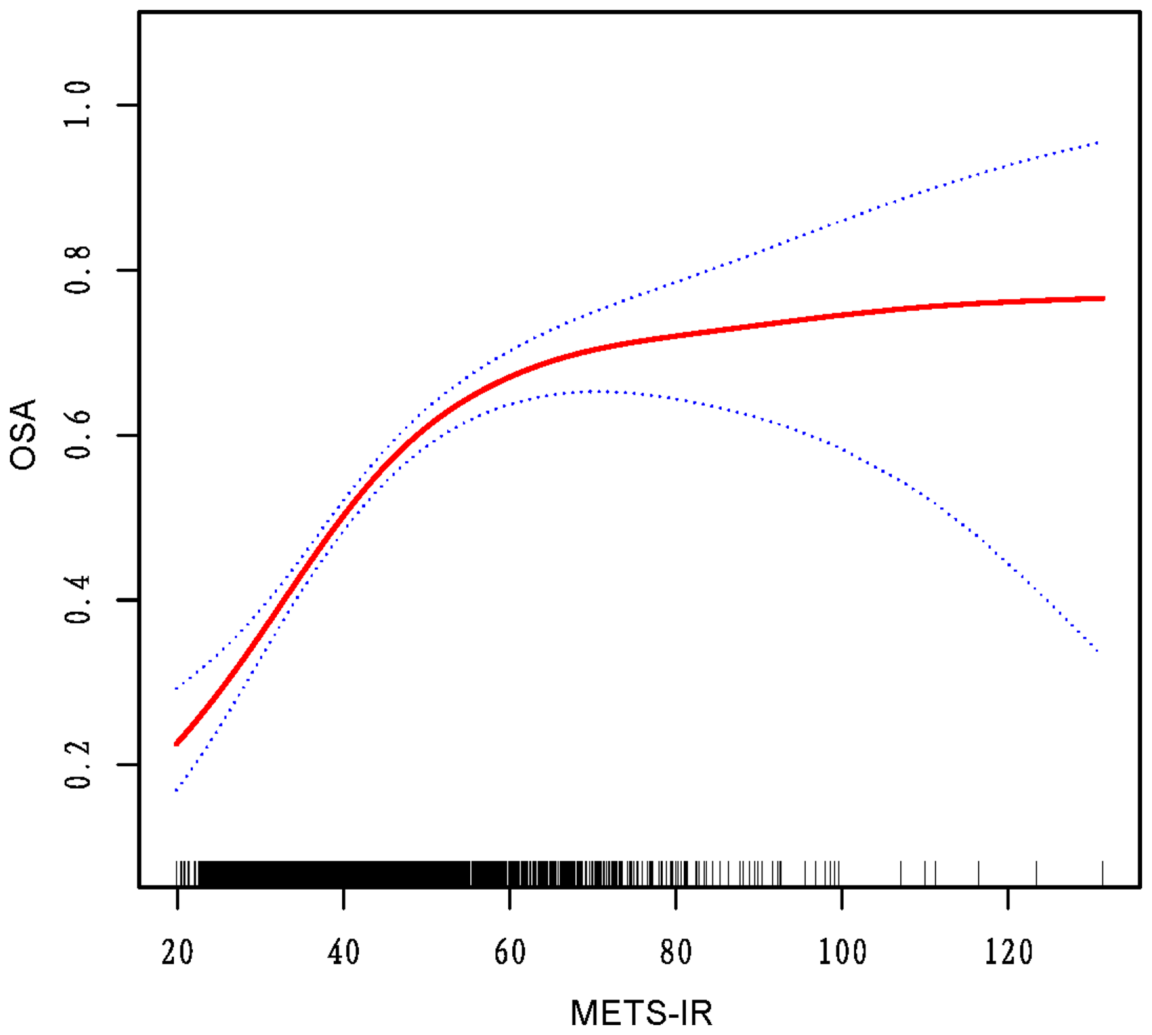
The red curve on the graph denotes the smoothed fit between the variables, with the blue lines outlining the 95% confidence intervals around the fit.

The analysis of threshold effects revealed a non-linear association between METS-IR and OSA risk (Figure 2). A significant increase in OSA risk was observed with METS-IR values under 46.65, with each unit increase correlating to a 7% rise in risk (OR = 1.07; 95% CI 1.05–1.08; *p* < 0.0001). Beyond this threshold, the rate of risk increase slowed to 1% (OR = 1.01; 95% CI 1.00–1.02; *p* = 0.0394). This finding was further corroborated by the likelihood ratio test (*p* < 0.001), suggesting a meaningful improvement in model fit (Table 3).

**Table 3:** Analysis of the threshold effect between METS-IR and the risk of OSA.

Threshold effect analysis	OSA
	OR (95%CI) *p*-value
Inflection point of METS-IR (K)	46.65
<K slope	1.07 (1.05, 1.08) <0.0001
>K slope	1.01 (1.00, 1.02) 0.0394
Log-likelihood ratio test	<0.001

METS-IR, metabolic score for insulin resistance; OSA, obstructive sleep apnea.

### Subgroup analyses

Subgroup analyses were undertaken to investigate the stability of the link between METS-IR and the risk of OSA, incorporating tests for interaction effects. In model 3, all potential confounders were included in the analysis alongside variables that defined the study subgroups. The results consistently indicated a stable relationship between METS-IR and various demographic factors, such as race, gender, age, education level, marital status, and socioeconomic status, along with physical activity, alcohol consumption, smoking habits, diabetes mellitus, hypertension, and cardiovascular conditions across all groups. No significant interaction effects were found since all interaction *p*-values exceeded 0.05 (Table 4).

**Table 4:** Subgroup analysis for the association between METS-IR and the risk of OSA.

Subgroup	OR (95%CI) *P*-value	P-interaction
Gender		0.4678
Male	1.04 (1.02, 1.06) 0.0008	
Female	1.05 (1.04, 1.07) <0.0001	
Age		0.7515
≤40	1.04 (1.01, 1.07) 0.0180	
>40, ≤60	1.05 (1.03, 1.07) 0.0009	
>60	1.05 (1.03, 1.07) 0.0007	
Race		0.6361
Mexican American	1.03 (1.01, 1.06) 0.0229	
Other Hispanic	1.05 (1.03, 1.08) 0.0039	
Non-Hispanic White	1.05 (1.03, 1.06) 0.0002	
Non-Hispanic Black	1.05 (1.03, 1.08) 0.0007	
Other Race	1.04 (1.02, 1.07) 0.0157	
Marital status		0.0668
Married	1.04 (1.03, 1.06) 0.0002	
Single	1.04 (1.02, 1.06) 0.0011	
With partner	1.07 (1.05, 1.10) 0.0003	
Education level		0.0798
Under high school	1.05 (1.04, 1.06) <0.0001	
High school or	1.03 (1.00, 1.05) 0.0539	
PIR		0.4761
<1.0	1.04 (1.01, 1.07) 0.0222	
1.0–3.0	1.04 (1.03, 1.06) 0.0002	
>3.0	1.05 (1.04, 1.07) 0.0001	
PA		0.8667
No	1.05 (1.03, 1.06) <0.0001	
Yes	1.05 (1.02, 1.07) 0.0023	
Alcohol use		0.0919
No	1.05 (1.04, 1.06) <0.0001	
Yes	1.03 (1.01, 1.05) 0.0296	
Smoking status		0.3426
No	1.05 (1.04, 1.07) <0.0001	
Yes	1.04 (1.03, 1.06) 0.0001	
Diabetes mellitus		0.5497
No	1.05 (1.03, 1.06) <0.0001	
Yes	1.04 (1.02, 1.06) 0.0021	
Hypertension		0.0857
No	1.05 (1.04, 1.07) <0.0001	
Yes	1.03 (1.01, 1.05) 0.0048	
CVD		0.2253
No	1.05 (1.04, 1.06) <0.0001	
Yes	1.03 (1.00, 1.06) 0.0524	

## Discussion

This study, leveraging data from the NHANES conducted between 2015 and 2018, seeks to elucidate the link between METS-IR and OSA. The results indicate a statistically significant positive link between METS-IR and the prevalence of OSA. Specifically, an increment of one unit in METS-IR is associated with a 5% increase in the probability of OSA (odds ratio [OR] 1.05, 95% confidence interval [CI] 1.04–1.06). This association remains significant even after accounting for various confounding factors. Potential confounding variables such as ethnicity, sex, age, and health-related aspects have been evaluated in our study. Significantly, participants categorized in the uppermost quartile of METS-IR demonstrate a risk of OSA that is approximately five times greater than that observed in those within the lowest quartile (OR: 4.95, 95% CI: 3.32–7.37). Furthermore, our investigation reveals a non-linear correlation between METS-IR and the risk of OSA, identifying a pivotal threshold at a METS-IR value of 46.65.

The intricate relationship between insulin resistance and OSA has attracted considerable attention in the fields of sleep medicine and metabolic research. This study contributes to an extensive body of literature that has largely focused on individual metabolic parameters, often overlooking the complex interactions between these two conditions. For example, Soo-Young Yoon has found a correlation between fasting blood glucose levels and an elevated risk of cardiovascular events (20). Moreover, insulin sensitivity has been linked with cardiovascular risk factors treated as continuous variables, as shown in the research by Laakso (21). The relationship between triglycerides, a marker for dyslipidemia, and insulin resistance has been systematically reviewed by Bjornstad and Eckel (22). Additionally, Schmiegelow et al. provided a comprehensive review of the association involving high-density lipoprotein cholesterol, recognized for its protective role against cardiovascular diseases, and insulin resistance (23). The association between body mass index, a widely recognized obesity indicator, and insulin resistance has been thoroughly explored in a study conducted by Abdelhamed et al. (24). In contrast to these studies that concentrate on isolated metrics, our research employs METS-IR, a composite index that integrates fasting blood glucose, triglycerides, BMI, and HDL-C, thereby effectively representing the overall metabolic profile linked to insulin resistance and OSA (15). Furthermore, Januzzi has emphasized the significance of multiple biomarkers in predicting cardiovascular events (25), which aligns with our approach of utilizing the METS-IR to assess the correlation between insulin resistance and OSA.

It is important to note that, as far as we are aware, this study is one of the first to explore the nonlinear link between METS-IR and OSA. It reveals a potential threshold effect, which offers new insights that could enhance clinical decision-making and risk assessment for OSA. The identified inflection point of METS-IR at 46.65 corresponds to approximately 60% of the total OSA risk, with the rate of risk increase slowing after this threshold, reaching an estimated 75% of OSA risk. This highlights the significant role of metabolic fitness in OSA risk, while also emphasizing the importance of other factors, such as obesity, age, and comorbidities like hypertension, which become more influential at higher risk levels (26–28).While METS-IR is an important factor, these findings suggest that OSA risk is shaped by a complex interplay of metabolic, lifestyle, and traditional risk factors. Future research should explore how these factors interact to refine our understanding of OSA risk. Besides, above the inflection point, adaptability mechanisms, such as increased physical activity, play a role in reducing OSA risk (29). Physical activity improves metabolic syndrome and reduces OSA risk, while low vitamin D levels (30), linked to both OSA and metabolic dysfunction, may further influence risk. Maintaining adequate vitamin D levels could mitigate OSA risk, especially in individuals with high METS-IR scores. However, further research is required to elucidate the precise role of these adaptations in modulating OSA risk at elevated METS-IR levels.

Regarding the mechanisms that may elucidate the link between METS-IR and OSA, it is feasible that various physiological and metabolic pathways are implicated. As a comprehensive measure of insulin resistance, METS-IR effectively reflects an individual’s metabolic health status. Specifically, insulin resistance is commonly associated with an abnormal accumulation of adipose tissue, particularly in the cervical and abdominal areas, which heightens the risk of OSA. The anatomical resistance of the airway is a significant factor contributing to the worsening of OSA (31, 32). Evidence indicates that an elevated METS-IR is closely associated with alterations in body fat distribution, potentially leading to mechanical obstruction in the airway (15, 33). Additionally, elevated METS-IR is often associated with the onset of chronic low-grade inflammation. Insulin resistance may trigger the release of inflammatory mediators such as tumor necrosis factor-alpha and interleukin-6, potentially compromising airway structural integrity and disrupting central nervous system functions. The association of respiration with OSA exacerbates the severity of the condition (34–36). Additionally, persistent inflammation can elevate oxidative stress levels, a factor that has been widely studied in relation to OSA, underscoring its critical importance in the pathophysiology of the disorder (37). The influence of OSA on sleep quality may perpetuate a negative cycle, as insufficient sleep and diminished sleep quality can lead to decreased insulin sensitivity, further intensifying metabolic dysregulation (38, 39). The rise in METS-IR not only signifies an increased level of insulin resistance but may also engage in a synergistic relationship with the worsening of sleep disturbances, resulting in a complex pathological mechanism. Furthermore, both genetic susceptibility and environmental factors, such as lifestyle habits and dietary choices, could significantly impact the relationship between METS-IR and OSA (39, 40). This finding underscores the necessity for subsequent research to explore the complex interactions between these elements. Ultimately, our study underscores the pivotal role of METS-IR as an indicator of insulin resistance in the development of OSA, stressing the imperative to improve metabolic health in the context of OSA management. The findings facilitate the development of early detection and intervention strategies for OSA. This study enhances our comprehension of the pathophysiological mechanisms associated with OSA and proposes potential intervention strategies for future clinical practice.

This research represents a significant advancement in the field by performing a systematic examination of the relationship between METS-IR—a composite measure encompassing various metabolic indicators—and OSA. It offers a fresh perspective on how metabolic health correlates with the risk of developing OSA. The nonlinear analysis identified a noteworthy nonlinear link between METS-IR and the risk of OSA, pinpointing a critical threshold at 46.65. This discovery lays a scientific groundwork for early detection and targeted interventions for OSA. Furthermore, the use of the NHANES database ensures a diverse and representative sample while complying with the established guidelines of the database.

However, this research has inherent limitations. Firstly, the cross-sectional design of this investigation could restrict the robustness of conclusions drawn about causality between exposure and outcome, highlighting the necessity for future longitudinal research to validate our findings Secondly, the diagnosis of OSA relies on self-reporting rather than objective methods like polysomnography, which may introduce biases, affecting the accuracy of results. Additionally, although we controlled for various covariates, the exclusion criteria did not encompass all potential sleep disorders that could cause excessive sleepiness, which may influence the interpretation of the results. Finally, the applicability of these findings to other populations is still unclear, indicating a need for further investigation.

## Conclusion

In conclusion, this investigation reveals a significant and nonlinear relationship between METS-IR and OSA. Future research should include prospective and randomized controlled studies to validate these findings. Additionally, further exploration of the pathophysiological mechanisms underlying these associations is essential.

## Data Availability

Publicly available datasets were analyzed in this study. This data can be found at: this data can be found at: NHANES (https://wwwn.cdc.gov/nchs/nhanes/default.aspx).

## Footnotes

^1^ https://www.empowerstats.net/cn/

^2^ https://www.r-project.org/
